# Chronic disease and falls in community-dwelling Canadians over 65 years old: a population-based study exploring associations with number and pattern of chronic conditions

**DOI:** 10.1186/1471-2318-14-22

**Published:** 2014-02-14

**Authors:** Kathryn M Sibley, Jennifer Voth, Sarah E Munce, Sharon E Straus, Susan B Jaglal

**Affiliations:** 1 Toronto Rehabilitation Institute, University Health Network, Toronto, Canada; 2 Department of Physical Therapy, University of Toronto, Toronto, Canada; 3 Department of Psychology, University of Windsor, Windsor, Canada; 4 Institute of Health Policy, Management and Evaluation, University of Toronto, Toronto, Canada; 5 Li-Ka-Shing Knowledge Institute, St. Michael’s Hospital, Toronto, Canada; 6 Department of Medicine, University of Toronto, Toronto, Canada

## Abstract

**Background:**

Falls and chronic disease are both important health issues in older adults. The objectives of this study were to quantify the prevalence of falls and multi-morbidity (≥2 chronic conditions) in Canadian older adults; examine associations between falls and number of chronic conditions; and explore whether certain patterns of chronic disease were associated with a greater risk of falling.

**Methods:**

Data were derived from the Canadian Community Health Survey- Healthy Aging. Primary outcomes from 16,357 community-dwelling adults aged 65 years and over were self-reported falls in the previous 12 months and presence of 13 chronic conditions. Prevalence estimates were calculated with normalized sampling weights, and hierarchical cluster analysis was used to identify clusters based on chronic condition patterns, and tested for association to falls with logistic regression.

**Results:**

Overall prevalence of falling and multi-morbidity were 19.8% and 62.0% respectively. Fall risk was significantly greater in individuals with one, two, four, five and six or more chronic conditions relative to those with none (all p < 0.05). A seven-cluster model was selected, including groups with low prevalence of chronic disease, or high prevalence of hypertension and arthritis, visual impairment, hypertension, chronic obstructive pulmonary disease (COPD), diabetes, or heart disease and hypertension. Only the hypertension cluster (Odds Ratio [OR] = 1.2) and COPD cluster (OR = 1.6) were significantly associated with increased falls relative to the low prevalence group.

**Conclusions:**

Both the number and pattern of chronic conditions were related to falls. COPD emerged as a significant predictor of falls despite affecting a smaller proportion of respondents. Continued study is warranted to verify this association and determine how to incorporate consideration of chronic disease and multi-morbidity into fall risk assessments.

## Background

Falls are a major health concern for older adults, often triggering a downward spiral in health that is associated with activity restriction [1], high health care costs [2] and long term care admission [3]. Many factors contribute to falls, and among these, a number of chronic conditions, including arthritis, diabetes and visual impairment, are associated with increased fall risk [4]. The falls literature has regarded such conditions as mediating factors, and when chronic diseases are considered, it is often in the context of the effect of a single condition. For example, a recent systematic review on fall risk factors identified at least six chronic conditions independently associated with falls in a minimum of two prospective cohort studies, however they were all treated as independent risk factors and there was no indication whether chronic conditions were considered in multiple or combination [4]. In fact, the emerging older adult population is increasingly complex, and chronic disease is on the rise [5]. Approximately 92% of older adults have at least one chronic disease [5] and 65–85% have two or more co-existing chronic diseases [6], commonly described as multi-morbidity [7]. Optimizing health service delivery for older adults requires a shift away from single-disease models of care [8]. However, current fall prevention guidelines [9] do not explicitly account for the role of chronic disease (either in single or multiple), and many of the practice guidelines for individual chronic diseases do not consider multi-morbidity or falls [10,11]. This may be due to the limited information on the association between total disease burden and falls from which to inform guidelines.

To date, only a few studies have investigated the association between multi-morbidity and falls [12-14]. For example, Lee et al. observed substantial co-occurrence of several chronic conditions and falls among 11,000 older adults, and increased prevalence of falling among those with a chronic disease than without [12]. In another study of 12,669 community-dwelling older adults receiving Medicare in the United States, Shumway-Cook et al. determined that the likelihood of having 1 or 2 or more falls in the previous year (relative to no falls) was associated with increasing number of comorbidities [13]. However, existing studies of falls and multi-morbidity have a number of limitations. First, any examination of “multi-morbidity” to date has been restricted to exploring the impact of simple disease counts on falls, and has not examined the influence of particular combinations of disease. While evidence to date suggests that number of chronic conditions is important, there may be an additional layer of importance related to falls as a function of the pattern of chronic conditions across individuals. Second, there has been extensive variation in both the number and type of chronic conditions considered across previous falls and multi-morbidity studies. Although there is no gold standard index of chronic disease, a recent systematic review on multi-morbidity indices recommended the inclusion of eight diagnoses based on overall prevalence in the population as a representative standard (arthritis, cancer, chronic obstructive pulmonary disease [COPD], diabetes, depression, heart disease or myocardial infarction, hypertension, stroke), as well as relevant or study-specific conditions [15]. Given the varied nature of aging and range of conditions associated with increased fall risk, a comprehensive approach to considering multi-morbidity in relation to falls is warranted.

Improved understanding of relationships between falls and chronic disease in older adults is needed to provide recommendations that will be of maximal use to clinicians who need to provide integrated care for complex patients. This study addressed gaps in the literature by exploring whether particular combinations of chronic disease were associated with falls in a recent Canadian population-based sample. Chronic disease patterns were investigated with cluster analysis, which facilitated exploration of the complex array of conditions comprising “multi-morbidity”, and whether particular patterns were associated with falls. The specific objectives were to quantify the prevalence of falls, individual chronic conditions and multi-morbidity in community-dwelling Canadians aged 65 years and older; examine associations between falls, individual chronic conditions, and number of chronic conditions; and explore whether clusters based on patterns of chronic conditions experienced elevated rates of falls.

## Methods

This study was approved by the Research Ethics Board at the University of Toronto. Secondary data analyses were conducted using the Canadian Community Health Survey – Healthy Aging, a population-based, cross-sectional survey conducted by Statistics Canada between December 2008 and November 2009. Details of the survey, including data sources and methodology, the questionnaire, and data accuracy are described online [16] and in a published report [17]. In brief, a multistage stratified cluster design sampling procedure based on the 2006 Census was used, and included people aged 45 or older living in private dwellings in all ten Canadian provinces. Individuals were excluded if they lived in the three territories, some remote regions, institutions, Indian reserves or Crown lands, military bases, or were full-time members of the Canadian Forces. Computer-assisted personal interviewing was used to collect information about general health and well-being, physical activity, use of health care services, social participation, work and retirement transitions. The survey was completed by 30,865 individuals; an overall response rate of 74.4%. Multiple quality control steps were used, including error detection by the computer-assisted interview software, validation with outcomes common in other Canadian population surveys, and external validation by federal and provincial partners.

This study includes data from 16,357 individuals aged 65 and over who answered the question, “in the past 12 months, did you have any falls?”. Data for the present study were derived from questions throughout the survey and were collapsed or recategorized where appropriate. The primary outcome for this study was self-reported falls in the previous twelve months (binary: yes/ no). Secondary variables related to falls included number of falls, injuries related to falls, need for medical attention and hospitalizations resulting from falls. As per recommendations for measuring multi-morbidity, we included eight frequently reported chronic diseases (arthritis, cancer, chronic obstructive pulmonary disease [COPD], diabetes, depression, heart disease [angina or myocardial infarction], hypertension, stroke) and five study-specific chronic conditions with known associations to falls or hip fracture (dementia, osteoporosis, Parkinson’s disease, urinary incontinence, and visual impairment [cataracts or glaucoma]) [4,18-20], all defined in the survey as lasting 6 months or more and diagnosed by a health professional. Presence or absence of each condition as well as total number of conditions was calculated. Individuals were defined as having multi-morbidity if they had two or more chronic conditions. Clinical and demographic characteristics included age, sex, marital status, geographic setting, living arrangement, mobility, activities of daily living impairment, fear of falling, use of four or more medications, use of psychotropic medications (tranquilizers, antidepressants or sleeping pills), fear of falling, and vitamin D supplementation.

Analysis was performed with SAS version 9.3 (SAS Institute, Cary, NC). To account for the stratified survey sampling design, normalized sampling weights were applied to prevalence estimates and bootstrap weights were applied to variance calculations, using data provided by Statistics Canada. Descriptive statistics were calculated for all variables. Proportional differences in outcome variables between groups were compared with the Rao-Scott X^2^ test. Binary logistic regression modeled risk of falling as function of number of chronic conditions.

To explore whether some chronic disease patterns had a greater influence on falls than others, a two-step procedure was applied. Cluster analysis was used to identify meaningful sub-groups of individuals based on their responses to the chronic disease questions in the survey (binary indicators) [21]. Chronic conditions with very low prevalence (< 2.0%) were excluded from cluster analysis to minimize chaining (sequential joining into clusters) [22]. We used a hierarchical agglomerative clustering approach in which all individuals in the dataset are first treated as their own clusters, and are then merged into larger groupings based on a series of steps that attempt to minimize the variance within clusters so that the members in each cluster are as similar to each other as possible and as different as possible from members in other groupings [22,23]. A hierarchical approach attempts to find the optimal solution when the true number of clusters is not known [22], and we used Ward’s minimum variance method [24], which defines the distance between clusters as the increase in sum of squares within clusters after each step and summed over all variables, to identify the optimal number of groupings among participants using the jaccard dissimilarity coefficient. Ward’s method was chosen because it performs well in simulation studies comparing clustering methods and because it tends to produce well-distributed clusters [22,25]. The appropriate number of clusters was decided by consulting each agglomeration schedule, noting local peaks in the pseudo-F statistic that corresponded with decreases in pseudo-t at each subsequent step, and examining common peaks across each method [26]. The dendogram for each method was also consulted. The final cluster solution was validated first by comparing the results to solutions generated by the average linkage and centroid hierarchical clustering methods – two complementary clustering methods that define the distances between clusters based on average distance between pairs of objects between clusters (average linkage) and squared Euclidean distance between mean vectors (centroid) [22] – and then by running separate cluster analyses on each half of the randomly divided dataset to investigate the stability of the final solution. Binary logistic regression was then used to determine whether any of the clusters were significantly associated with falls (controlling for age and gender as covariates). Odds ratios (ORs) are reported for significant associations.

## Results

### Prevalence of falls, individual chronic conditions and multi-morbidity

Selected clinical and demographic characteristics are presented in Table 1. The overall proportion of individuals who reported falling in the previous year was 19.8%. Detailed fall characteristics are presented in Table 2. In summary, among the fallers, 63.3% fell once in the previous year, while 36.7% fell more than once. Thirty-five percent reported no serious injury as a result of their fall(s). Among those who experienced some injury, 29.3% sought medical attention, and 7.8% reported being hospitalized. Sixty-two percent of respondents had multi-morbidity, while 23.8% of respondents had a single chronic condition and 14.2% had no chronic condition. The prevalence of specific individual chronic conditions is presented in Table 3. All thirteen chronic conditions considered were observed.

**Table 1:** Demographic and clinical characteristics

**Variable**	**Total Sample (weighted %)**	**Fallers (weighted %)**	**Non-fallers (weighted %)**	**Rao-Scott X**^**2**^**(weighted & boot-strapped)**	**P**	**Fallers 95% CI for proportion**	**Non-fallers 95% CI for proportion**
Age (years)				63.4	<0.0001		
65-74	55.2	47.3	57.1			44.9-49.7	56.5-57.7
75-84	33.6	37.4	32.7			35.3-39.5	32.2-33.2
85+	11.2	15.3	10.2			14.0-16.7	9.9-10.5
Sex				28.2	<0.0001		
Male	45.1	38.8	46.7			36.5-41.1	46.1-47.3
Female	54.9	61.2	53.3			58.9-63.5	52.7-53.9
Married				41.2	<0.0001		
Yes	63.4	56.1	65.2			53.5-58.7	64.0-66.3
No	36.0	43.9	34.8			41.3-46.4	33.7-36.0
Living arrangement				24.4	<0.0001		
Alone	29.3	34.5	28.0			32.1-36.9	26.8-29.2
With others	64.8	60.4	65.9			57.9-62.8	64.7-67.1
Other	5.9	5.1	6.1			3.9-6.4	5.25-7.01
Geographic setting				0.04	0.84		
Urban	78.5	78.3	78.6			75.3-81.4	76.4-80.8
Rural	21.5	21.7	21.4			18.6-24.7	19.2-23.6
Mobility				258.7	<0.0001		
Walks independently	87.5	76.1	90.3			74.2-78.1	89.6-91.1
Walks with aid or support	11.7	22.3	9.1			20.4-24.3	8.4-9.9
Cannot walk	0.7	1.5	0.5			0.9-2.1	0.4-0.7
ADL impairment				238.4	<0.0001		
None	77.4	63.6	80.8			61.2-66.1	79.7-81.8
Mild/Moderate	20.1	30.9	17.4			28.5-33.3	16.4-18.4
Severe/Total	2.6	5.5	1.8			4.4-6.6	1.5-2.6
Medication use				106.1	<0.0001		
>4	85.0	76.5	87.1			74.5-78.6	86.1-88.0
<=4	15.0	23.5	12.9			21.5-25.5	12.0-13.7
Fear of falling				188.3	<0.0001		
Yes	33.6	49.6	29.7			46.9-52.2	28.4-31.0
No	66.4	50.4	70.3			47.7-53.1	69.0-71.6
Psychotropic medications				75.2	<0.0001		
Yes	19.4	27.6	17.4			25.3-29.9	16.4-18.4
No	80.6	72.4	82.6			70.1-74.7	81.6-83.6
Vitamin D supplementation		42	38	7.8	0.0052		
Yes	39.4	.8	.6			40.1-45.5	37.4-39.9
No	60.6	57.2	61.4			54.5-59.9	60.1-62.6

ADL = Activities of daily living.

**Table 2:** Fall characteristics

**Variable**	**Weighted %**
Number of falls	
1	63.3
2	19.0
3	8.1
4	3.7
> = 5	5.4
Most serious injuries resulting from fall(s)	
No serious injury	35.1
Bruises	21.2
Sprain/strain	9.9
Discomfort	8.6
Other	8.1
Cuts	5.6
Fracture of arm or wrist	3.5
Head injury	3.0
Fracture of leg	2.3
Fracture of back/vertebra	1.4
Fracture of hip	1.2
Medical attention sought	
Yes	29.3
No	70.7
Hospitalization after fall	
Yes	7.8
No	91.0

**Table 3:** Chronic conditions and falls

**Condition**	**Prevalence (weighted %)**	**% of people with condition who fell (weighted %)**	**% of people without condition who fell (weighted %)**	**Rao-Scott X**^**2 **^**(weighted & boot-strapped)**	**P value**	**95% CI for % of people with condition who fell**	**95% CI for % of people without condition who fell**
Hypertension	50.2	21.4	18.3	10.8	0.01	20.1-22.7	16.9-19.6
Arthritis	43.4	24.4	16.3	82.9	< 0.0001	22.9-25.9	15.1-17.4
Vision impairment	27.8	24.1	18.2	32.6	< 0.0001	22.3-25.9	17.1-19.3
Heart disease	22.6	24.4	18.5	30.3	< 0.0001	22.5-26.4	17.5-19.6
Osteoporosis	18.1	25.5	18.5	36.8	< 0.0001	23.2-27.7	17.5-19.5
Diabetes	17.2	23.8	19.0	17.1	< 0.0001	21.6-26.1	18.0-20.0
Incontinence	11.7	28.1	18.7	49.7	< 0.0001	25.4-30.7	17.7-19.8
COPD	8.8	25.5	19.3	18.1	< 0.0001	22.4-28.6	18.3-20.2
Cancer	5.5	22.9	19.6	2.2	0.14	18.4-27.4	18.7-20.6
Depression	5.1	36.0	19.0	52.4	<0.0001	31.0-41.1	18.0-19.9
Stroke	4.2	36.8	19.1	72.9	< 0.0001	32.0-41.6	18.1-20.0
Dementia	1.6	38.5	19.5	31.3	< 0.0001	30.4-46.6	18.6-20.5
Parkinson’s disease	0.8	30.8	19.8	4.7	0.029	19.5-42.1	18.8-20.5

COPD = Chronic obstructive pulmonary disease.

### Associations between falls, individual chronic conditions and number of chronic conditions

Falls in the previous twelve months were significantly lower among people with no chronic conditions (11.4%) compared to those with any chronic condition (21.2%, X^2^ [1] = 60.0, p < 0.0001); fall rates ranged from 21.4-38.5% in those with a chronic condition (Table 3). Risk of falling increased as a function of the number of chronic conditions (Table 4), and was significantly elevated among individuals with one, two, four, five and six or more chronic conditions relative to those with no chronic conditions (all p < 0.05).

**Table 4:** Logistic regression results for number of chronic conditions and falls

**Number of chronic conditions**	**X**^**2**^	**P value**	**OR**
0	Reference		
1	9.1	0.03	1.3
2	3.7	0.05	1.4
3	0.1	0.7	1.7
4	5.1	0.02	2.0
5	4.6	0.03	2.1
6 or more	13.4	0.003	2.7

### Influence of specific chronic disease patterns on falls

Dementia and Parkinson’s Disease were omitted from the cluster analysis due to low prevalence (< 2% each). Ward’s method suggested that a seven-cluster solution best fit the data, which was supported by visual inspection of the dendogram (Additional file 1). The seven-cluster solution was validated by replication with the centroid method, while the average linkage method suggested a highly similar cluster pattern. When the data were split into two random halves, Ward’s method also indicated that a seven-cluster solution fit both halves. As such, the robust seven-cluster pattern was selected for the final solution.

Prevalence of each chronic condition, clinical and demographic characteristics are presented by cluster in Table 5. All chronic conditions were represented in each cluster, although each cluster was dominated by a high prevalence (>70%) of one or two conditions, or in one case: relatively low prevalence of all conditions. As such, cluster #1 (28.8% of sample) was labeled “low chronic disease”; cluster #2 (12.3%): “hypertension and arthritis”; cluster #3 (12.6%): “visual impairment”; cluster #4 (19.6 %): “hypertension”; cluster #5 (5.7%): “COPD”; cluster #6 (11.6%): “diabetes”; and cluster #7 (9.4%) was labeled “heart disease and hypertension”.

**Table 5:** Prevalence of chronic conditions, demographic and clinical characteristics by cluster

**Cluster Number**	**1**	**2**	**3**	**4**	**5**	**6**	**7**
**Label**	**Low chronic disease**	**Hypertension and arthritis**	**Visual impairment**	**Hypertension**	**COPD**	**Diabetes**	**Heart disease and hypertension**
Weighted % with condition							
Arthritis	33.5	70.6	46.0	35.8	59.6	45.1	38.7
Osteoporosis	3.8	29.4	1.1	12.6	16.2	8.5	1.8
Hypertension	4.4	100.0	51.6	96.8	51.7	61.8	84.5
Diabetes	0.6	8.2	6.3	2.6	18.8	100.0	22.6
Stroke	0.9	5.9	1.6	8.9	5.1	5.7	2.8
COPD	2.1	5.9	2.6	1.6	99.1	4.0	7.6
Urinary incontinence	12.0	12.6	8.1	13.6	14.3	14.9	5.3
Vision impairment	4.2	37.4	89.4	9.5	38.9	21.7	44.1
Heart disease	16.5	23.8	2.6	3.8	30.0	23.3	100.0
Cancer	8.8	0.9	1.4	4.9	5.5	8.3	4.1
Depression	0.9	5.7	17.6	1.3	7.9	7.8	3.2

COPD = Chronic Obstructive Pulmonary Disease.

Selected clinical and demographic characteristics are presented for each cluster in Table 6. Relative to cluster #1 (“low chronic disease”), the remaining clusters tended to be older, female, require more mobility assistance, have greater impairment in activities of daily living, and took more medications (p < 0.01). Logistic regression results revealed that only clusters #4 (hypertension) and #5 (COPD) were significantly associated with increased risk of falls relative to cluster #1 (OR = 1.2, p = 0.048 and OR = 1.6, p = 0.03, respectively; Table 7).

**Table 6:** Clinical and demographic conditions by cluster

**Cluster Number**	**1**	**2**	**3**	**4**	**5**	**6**	**7**	**Rao-Scott X**^**2 **^**(weighted & boot-strapped)**	**P value**
**Label**	**Low chronic disease**	**Hypertension and arthritis**	**Visual impairment**	**Hypertension**	**COPD**	**Diabetes**	**Heart disease and hypertension**		
Age (years)								148.3	< 0.0001
65-74	62.6	46.9	49.3	56.4	52.3	58.5	46.0		
75-84	27.5	37.7	37.9	33.9	35.7	33.4	39.4		
85+	9.9	15.4	12.8	9.7	12.0	8.1	14.3		
Sex								486.4	< 0.0001
Male	53.4	13.6	41.9	42.6	46.7	54.6	58.3		
Female	46.6	86.4	58.1	57.4	53.3	45.4	41.7		
Married								98.9	< 0.0001
Yes	67.8	51.0	61.1	66.5	63.2	65.0	60.8		
No	32.2	49.0	38.9	33.5	36.8	35.0	39.2		
Living arrangement								63.3	< 0.0001
Alone	27.0	37.2	32.2	26.9	30.8	25.9	30.0		
With others	68.2	55.5	62.1	67.1	64.4	65.9	64.0		
Other	4.8	7.2	5.7	6.0	4.8	8.2	6.0		
Geographic setting								15.8	0.015
Urban	78.7	82.2	80.4	77.9	76.0	77.9	74.6		
Rural	21.3	17.8	19.6	22.1	24.0	22.1	25.4		
Current mobility status								210.6	< 0.0001
Walks independently	92.5	81.2	89.9	89.1	78.4	83.2	85.1		
Walks with aid or support	7.1	18.1	9.6	10.3	19.6	15.1	14.5		
Cannot walk	0.4	0.7	0.5	0.6	2.0	1.7	0.4		
Current ADL impairment status								153.2	< 0.0001
None	84.0	67.9	77.8	79.1	68.0	74.8	74.1		
Mild/Moderate	14.3	29.4	20.0	18.5	26.6	21.9	23.8		
Severe/Total	1.7	3.7	2.2	2.4	5.4	3.2	2.1		
Current number of medications								408.8	< 0.0001
<4	94.4	80.5	85.7	88.0	69.3	80.1	70.1		
> = 4	5.6	19.5	14.3	12.0	30.7	19.9	29.9		
Fear of falling								191.1	< 0.0001
Yes	24.8	48.3	37.6	31.4	39.6	36.6	33.9		
No	75.2	51.7	62.4	68.6	60.4	63.4	66.1		
Psychotropic medications								75.9	< 0.0001
Yes	14.2	25.4	23.8	17.6	28.0	19.6	22.3		
No	85.8	74.6	76.2	82.4	72.0	82.4	77.7		
Vitamin D supplementation								7.8	0.0052
Yes	35.5	63.5	40.4	39.0	38.1	31.6	30.0		
No	64.5	36.5	59.6	61.0	61.9	68.4	70.0		

ADL = Activities of daily living.

**Table 7:** Logistic regression results for chronic condition clusters and fall risk*

**Cluster**	**Label**	**X**^**2**^	**P**	**Odds Ratio**
#1	“Low chronic disease”	Reference		
#2	“Hypertension and arthritis”	0.1	0.8	1.3
#3	“Visual impairment”	1.6	0.22	1.2
**#4**	**“Hypertension”**	**3.9**	**0.048**	**1.2**
**#5**	**“COPD”**	**4.9**	**0.03**	**1.6**
#6	“Diabetes”	2.1	0.1	1.5
#7	“Heart disease and hypertension”	3.7	0.06	1.3

*Controlling for age and gender.

COPD = Chronic Obstructive Pulmonary Disease.

## Discussion

This study demonstrated that fall incidence among community-dwelling older Canadians is highly linked to the presence of chronic disease. Fall rates were significantly higher among individuals with a chronic condition compared to those without, and all individual chronic conditions with the exception of cancer were associated with increased prevalence of falls. Importantly, both number and type of chronic condition played a role in falls. With respect to number, fall rates increased linearly with the number of conditions. With respect to type, when considering combinations of chronic disease identified by cluster analysis, though some conditions were relatively evenly distributed across all clusters (e.g. cancer) and others were concentrated primarily in one cluster (e.g. diabetes), two groups of individuals were associated with increased risk of falls relative to the group with low prevalence of chronic disease: “hypertension” and “COPD”.

The finding that falls were related to the total disease burden supports previous studies showing increased risk of falling with multi-morbidity [12,13]. In particular, the observed linear trend between the prevalence of falling and number of chronic conditions substantiates the results of Lawlor et al. [14], who observed a linear relationship between the prevalence of falling in the previous year and the number of simultaneously present chronic diseases among community-dwelling women aged 60-79 years. Collectively, these results suggest an additive effect of chronic disease on fall risk, irrespective of the specific condition. While such information has some clinical value, the inability to speak to the influence of specific conditions is a limitation. The cluster analysis approach is a key strength of the current study, as it facilitated the exploration of the question about whether particular chronic disease combinations were associated with falls. Indeed, while our cluster analysis suggested that Canadian community-dwelling adults aged 65 years and older comprised seven distinct clusters based on chronic disease status, logistic regression revealed that not *all* of them were significantly associated with falls.

Interestingly, the dominating conditions in each of the two clusters significantly associated with falls – hypertension and COPD – were included in our consideration of multi-morbidity as a result of multi-morbidity measurement recommendations [15], not because of their specific association to falls. And yet, our finding that these clusters were associated with a significant increase in fall risk is supported by the literature. With respect to hypertension, there are several potential linking mechanisms to falls related to both the condition itself and treatment side effects known to induce orthostatic hypotension. Thiazide, prescribed as an anti-hypertensive medication, was associated with increased fall risk in the first three weeks after prescription in one prospective study of 56,000 older adults in primary care [27,28]. Emerging evidence also suggests that hypertension itself is a risk factor for orthostatic hypotension [29] and is related to falls. A recent prospective population study of 766 community-dwelling older adults determined that people with uncontrolled hypertension and orthostatic hypotension were 2.5 times more likely to have recurrent falls than those with uncontrolled hypertension and no orthostatic hypotension [30]. Similarly, fall risk has not traditionally been the primary concern for people with COPD, but new evidence demonstrates that this population has both increased prevalence of falls [31,32] as well as dysfunction in physiological risk factors for falls, such as impaired postural control [33]. While the specific mechanisms through which COPD acts on falls are not fully understood, skeletal muscle dysfunction and cerebral hypoxemia have been postulated as contributing factors [32]. We also note that while hypertension was the most prevalent chronic condition overall (50.2%) and the “hypertension” cluster was second in size only to the “low chronic disease” group, COPD prevalence was 8.8% overall and the “COPD” cluster was the smallest of the seven. As such, although COPD affected a relatively small proportion of individuals, the fact that it still emerged as a significant predictor of falls suggests it had a major impact on these people with respect to fall risk. That said, it is important to note that most chronic conditions individually had an effect on falls, and even though some may not have emerged as key drivers of chronic disease clusters across the population (stroke, for example), the fall risk associated with those conditions should not be understated.

Our study has a number of implications for clinical practice. First, given the differences in fall rates between individuals with and without chronic disease, the data underline differences within the community-dwelling older adult population with regard to falls, and that both the presence of specific chronic diseases and the number of conditions affecting an individual contribute to fall risk. Although current fall prevention guidelines [9] do not explicitly account for the role of chronic disease or multi-morbidity, clinicians may find this useful to consider when adapting these guidelines for individual patients. Second, the inherent challenges associated with managing multiple conditions require complementary strategies, and self-management approaches for older adults may offer additional opportunities to promote function and independence [34]. Although falls are episodic in nature, fall prevention requires sustained effort – similar to chronic disease management strategies – and many behaviors, such as exercise and medication monitoring, can benefit multiple conditions.

Many of the trends observed in this Canadian population-based analysis were lower than previous reports. The total fall prevalence rate of 19.8% was slightly lower than a similar American population-based retrospective study that reported falls among 22% of older adults on Medicare living in the community [13]. Taken together, the mounting population-based data suggests that the commonly reported statistic that one in three older adults fall each year [35-37] may no longer be accurate. The prevalence of chronic disease in the present sample was also lower than other studies, occurring in 76% of older adults and multi-morbidity occurring in 62%. In contrast, American data estimates chronic disease in over 90% of community-dwelling older adults [5], and our observed multi-morbidity rate was lower than the range reported in a recent systematic review of aging with multi-morbidity [6]. Although it could be interpreted that the present dataset may constitute a healthier population, between-study variations in the number of and specific chronic conditions considered may have contributed to some of these differences [38]. Comparison of chronic disease clusters between the current and previous analyses is also limited by the variation in populations and conditions considered across studies. While our analysis suggested that seven clusters best fit the data, Marengoni et al. [39] identified five clusters of multi-morbidity patterns in community-dwellers aged 77 years and over. While we are not aware of any other studies attempting to relate specific chronic disease patterns to falls in older adults, Vu et al. [40] explored chronic disease clusters in community-dwelling adults aged 65 and over who were hospitalized for a fall and identified five clusters. Unfortunately, it is not possible to make between-study cluster comparisons as all studies considered different numbers and type of chronic conditions.

Fall rates for many of the individual chronic conditions were also lower than previous reports (i.e. arthritis [41], visual impairment [18], diabetes [42], COPD [31], Parkinson’s disease [19], cancer [43], and urinary incontinence [20]), although variations in fall reporting methods, diagnostic criteria and disease severity may have accounted for this. Interestingly, we were unable to locate any peer-reviewed literature describing fall rates in people with osteoporosis. While many papers have discussed increased fall risk and the implications for hip fracture in this population, there appears to be a need for additional definitive research in this area.

Although the population-based analysis is a strength of this study, there are a number of limitations. As the survey was cross-sectional, cause-and-effect cannot be determined. All variables were derived from self-report data, not objective measures. Falls were identified by retrospective recall over the previous twelve months. Although other methods of fall reporting are more accurate [44,45], 12-month recall is representative of clinical practice and is the method advocated for assessing fall risk in published fall prevention guidelines [9]. Falls were not defined for survey participants, which could have variations in interpretation of what constitutes a fall, and additional inaccuracies in the self-report data. The fear of falling variable was limited to a dichotomous question, which has been criticized for lacking detail and sensitivity [46]. A limitation common across multi-morbidity studies is the arbitrary selection of conditions included, and given the nature of the secondary analysis, we were not able to apply more standardized classifications of multi-morbidity [47]. The survey did not include evaluation of some key fall risk factors (such as balance), the use of particular medications and only considered current mobility status, disability, medication use and living arrangement (which may or may not have been the same at the time of the fall), so we were unable to adjust for these factors in our model.

## Conclusions

These data illustrate the complex interplay between chronic disease and falls in older adults, highlighting the need for coordinated management of these health issues. While additional study is necessary to corroborate these findings, clinicians may consider multi-morbidity, hypertension, and COPD as particular ‘red flags’ for fall risk. Continued work is required to consider if and how chronic disease should be incorporated into fall prevention guidelines. In light of the emerging quantity and complexity of the aging population, collaborative efforts are required to optimize evidence-based care models of health service delivery for these individuals.

## Abbreviations

COPD: Chronic obstructive pulmonary disease; OR: Odds ratios.

## Competing interests

The authors declare that they have no competing interests.

## Authors’ contributions

KMS conceived of the study, designed the study, obtained ethics approval, conducted the analysis and wrote the manuscript. JV participated in study design, data analysis and interpretation, and manuscript writing. SEM participated in study design, data analysis and interpretation, and manuscript writing. SES participated in data analysis and interpretation and contributed to manuscript writing. SBJ participated in study conception, design, analysis interpretation and manuscript writing. All authors read and approved the final manuscript.

## Pre-publication history

The pre-publication history for this paper can be accessed here:

http://www.biomedcentral.com/1471-2318/14/22/prepub

## Supplementary Material

Additional file 1 **Dendogram illustrating the seven-group cluster solution selected for the present study (circles), using Ward’s minimum variance method.** The dendogram illustrates relationships of dissimilarity (reflected by the semi-partial r-squared of the Jaccard dissimiliarity coefficient, vertical axis) from 16,357 individuals (horizontal axis) based on their patterns of binary response to eleven self-reported chronic conditions (excluding Parkinson’s disease and Dementia). Click here for file
