# Supplementary material for: Chronic disease and falls in community-dwelling Canadians over 65 years old: a population-based study exploring associations with number and pattern of chronic conditions
Source: BMC Geriatr. 2014 Feb 14;14:22. doi: 10.1186/1471-2318-14-22 (PMC3928582; doi:10.1186/1471-2318-14-22)
Supplement: Additional file 1 — Dendogram illustrating the seven-group cluster solution selected for the present study (circles), using Ward’s minimum variance method. The dendogram illustrates relationships of dissimilarity (reflected by the semi-partial r-squared of the Jaccard dissimiliarity coefficient, vertical axis) from 16,357 individuals (horizontal axis) based on their patterns of binary response to eleven self-reported chronic conditions (excluding Parkinson’s disease and Dementia). [file 1471-2318-14-22-S1.docx]

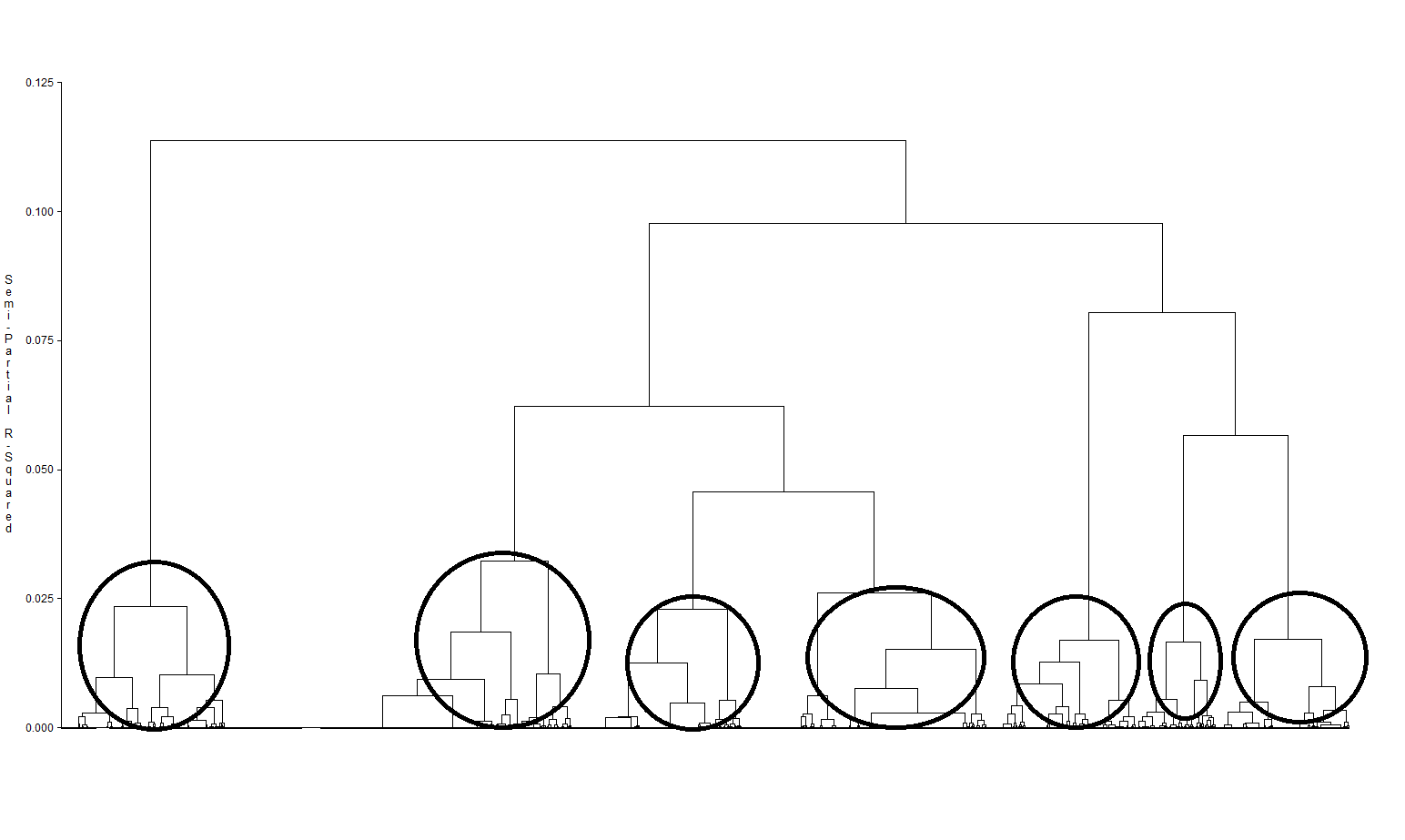


**Figure**. Dendogram illustrating the seven-group cluster solution selected for the present study (circles), using Ward’s minimum variance method. The dendogram illustrates relationships of dissimilarity (reflected by the semi-partial r-squared of the Jaccard dissimiliarity coefficient, vertical axis) from 16,357 individuals (horizontal axis) based on their patterns of binary response to eleven self-reported chronic conditions (excluding Parkinson’s disease and Dementia).
